# Supplementary material for: Patterns of Intron Gain and Loss in Fungi
Source: PLoS Biol. 2004 Nov 30;2(12):e422. doi: 10.1371/journal.pbio.0020422 (PMC532390; doi:10.1371/journal.pbio.0020422)
Supplement: Table S1 — Also available at http://genes.mit.edu/NielsenEtAl/. (4.3 MB ZIP). [file pbio.0020422.st001.zip › NielsenEtAl/html/738.html]

AN0086.1.NCU05680.1.MG01140.1.FG02714.1


```
 CLUSTAL W (1.82) Multiple Sequence Alignments - Introns Inserted


Sequence 1: NCU05680.1	503 aa
Sequence 2: MG01140.1	516 aa
Sequence 3: AN0086.1	498 aa
Sequence 4: FG02714.1	508 aa
Alignment Length: 519 aa
Number Identitical Residues: 317 aa
Alignment Score (without introns) 15364


MG01140.1 	MDLFRRARRLVPQQTPAQLPSAS-EKSKPQGPNRLTSRLAFFRRPLRLRGNSSVSVPLGV
NCU05680.1	MDLLRQLGGLLPGGRPPQLPLGQGEKDKPR--RTLAQRLQYLRRPLRLRGNSSISVPLGV
FG02714.1 	MDLIRRATRVAPSKPPLQLPLDEKRDSKHN--KAVDNRMAFFRRPLRLKGNSTISVPLGV
AN0086.1  	MGVVQRVRKWLPSTPSLPLDDSPYEKGHKS------SRFAFFRRRIRLKGNSSVSIPLGF
          	*.:.::     *   .  *     ...:        .*: ::** :**:***::*:***.

MG01140.1 	VILFPCIVVILILVLFVRHPSSPGR-ILMPAGAPPAIR2KISEKHDKVFVNGCLDPKTEA
NCU05680.1	VILFPCIVVILILVLFVRHPSSPGR-ILMPAGAPPGIR2KISEKYDKVFVTGCLEPDTSK
FG02714.1 	VIFFPMLVLILIFILFVSHPSSPGRGFLIPGGAAPKMR2KISEKYDKVFVEGCAQPDTSK
AN0086.1  	VLLFPCIVIVLILLLFVRHPSSPGG-ILIPAGTPPSIR2KISEKYDKVFATGCQPINTDT
          	*::** :*::**::*** ******  :*:*.*:.* :* *****:****. **   .*. 

MG01140.1 	DKAPRANAAFVVLARNKELDGVIQSIKSIERHFNRWFHYPYVFLNNGDFNETFKETVRNH
NCU05680.1	---PRANGAFVVLARNKELDGVIQSIKSIERHFNRWYHYPYVFLNDGDFDDTFKDTVKNY
FG02714.1 	---PRANAAFVILARNKELDGVLQSIKSVERHFNRWYNYPYVFLNDGDFDENFKEAIRNH
AN0086.1  	---PRANAAFVVLARNKEIDGVIQSMKSIERHFNRWYRYPYVFLNDAEFDDNFINTVKNY
          	   ****.***:******:***:**:**:*******:.*******:.:*::.* ::::*:

MG01140.1 	TSAPVEFGFVGPDMWGYPDWIDEKVAKEGIAKQGDAAVMYGGLESYHSMCRFYSG2FFYK
NCU05680.1	TSAPVEFGKVGPDMWGFPDWVDPKVAKEGIAKQGDAAVMYGGMESYHSMCRFYSG2FFYK
FG02714.1 	TSGEVEFGKVGPDMWGFPDWIDPKVVKEGIAKQGDAAVMYGGLESYHKMCRFYSG2FFFN
AN0086.1  	TSAPVEFGKIDETMWGYPSWVDHEVAKEGIRKQGDAAIMYGGMESYHHMCRFYSG2FFYK
          	**. **** :.  ***:*.*:* :*.**** ******:****:**** ******* **::

MG01140.1 	HPLLAKYEWYWRLEPEIKYFCDITY2DPFLKMIESNKTYGFTIAVKELRETVPNIFRYAS
NCU05680.1	HELLQKYEWYWRLEPEIKYFCDITY2DPFLKMIENNKTYGFTIAVKELRETVPNIFRYAS
FG02714.1 	HPLLLKYEWYWRVEPEISYFCDITY2DPFQKMIEHNKTYGFTIAVKELRETVPNIFRYAS
AN0086.1  	HPLLLKYEWYWRLEPEIKYFCDITY2DPFLKMAEANKTYGFTIAVKELRETVPNIFRYAS
          	* ** *******:****.******* *** ** * *************************

MG01140.1 	AYKRLKNITTQGLWEMFVEPTQEKEPEKEQLPEDDPNYKPPLPEEVTRNDPNRNTPIEID
NCU05680.1	AYKRLNNITSQGLWEMFVEPQPEKKPE---APE------PGLPEEILRSDPGANKLPDID
FG02714.1 	AYKRLNNITSKGLWEMFVEPPEDEEEDSPKESD------TSPNDNSFWDTLTGKKKNTID
AN0086.1  	AYKRKNNIKSQGLWEMFLEPREEPNPE-EQQPE-------TLPEEILITEPGEKN--EID
          	**** :**.::******:**  : : :  . .:          ::        :.   **

MG01140.1 	PEAMEGEKYNMCHFWSNFEIARLSWFRSKEYEDFFQMMDRSGGFWMER0WGDAPIHSLAA
NCU05680.1	PEAMEGEKYNMCHFWSNFEIARLDFFRSKAYEDFFQMMDHSGGFWMER0WGDAPIHSLAA
FG02714.1 	PESMEGEAYNMCHFWSNFEIAKLSWFRSKEYQDFFEMMDRSGGFWMER0WGDAPIHSLAA
AN0086.1  	PETMEGESYNMCHFWSNFEIARLDFFRSKEYEDFFEMMDRSGGFWMER0WGDAPIHSLAA
          	**:**** *************:*.:**** *:***:***:******** ***********

MG01140.1 	GVLLAPRDIHYFRDFGYRHTTIQHCPANAPSRQLAREPFLEKTTLDERKRHEEDQYWEQF
NCU05680.1	GALLGPRDIHYFRDFGYRHTTIQHCPANAPARQLPREPYLEKTTLDEKKRIEEDKYWDEY
FG02714.1 	GALLGPQDIHYFRDFGYRHTTIQHCPANAPGKQLAREPYLEKTTFPEYKRFEEDDYWEQW
AN0086.1  	GVLLSPSDIHYFRDFGYRHTTIQHCPANAPARQLPRIPYLEMTTEDEKERIEEDEYWANP
          	*.**.* ***********************.:**.* *:** **  * :* ***.** : 

MG01140.1 	DAPKENGVGCRCRCDTDIVDVEGKDGSCLAEWVDVAGGWASP-
NCU05680.1	DTPKENGVGCRCRCDTDIVDVEGKEGSCLAEWVDVAGGWASP-
FG02714.1 	DDVQEGGIGCRCRCDTDVVDVEGKEGSCLAEWVDVAGGWAHPY
AN0086.1  	DPVKENGVGCRCRCDTDIVDVEGKQGSCLAEWVEVAGGWASP-
          	*  :*.*:*********:******:********:****** *
```
